# Supplementary material for: Methionine restriction constrains lipoylation and activates mitochondria for nitrogenic synthesis of amino acids
Source: Nat Commun. 2023 May 2;14:2504. doi: 10.1038/s41467-023-38289-9 (PMC10154411; doi:10.1038/s41467-023-38289-9)
Supplement: Supplementary file 1 — Supplementary Information [file 41467_2023_38289_MOESM1_ESM.pdf]

Supplementary information

Methionine restriction constrains lipoylation and activates mitochondria for nitrogenic synthesis of amino acids

Wen Fang, Liu Jiang, Yibing Zhu, Sen Yang, Hong Qiu, Jiou Cheng, Qingxi Liang, Zong-cai Tu, and Cunqi Ye

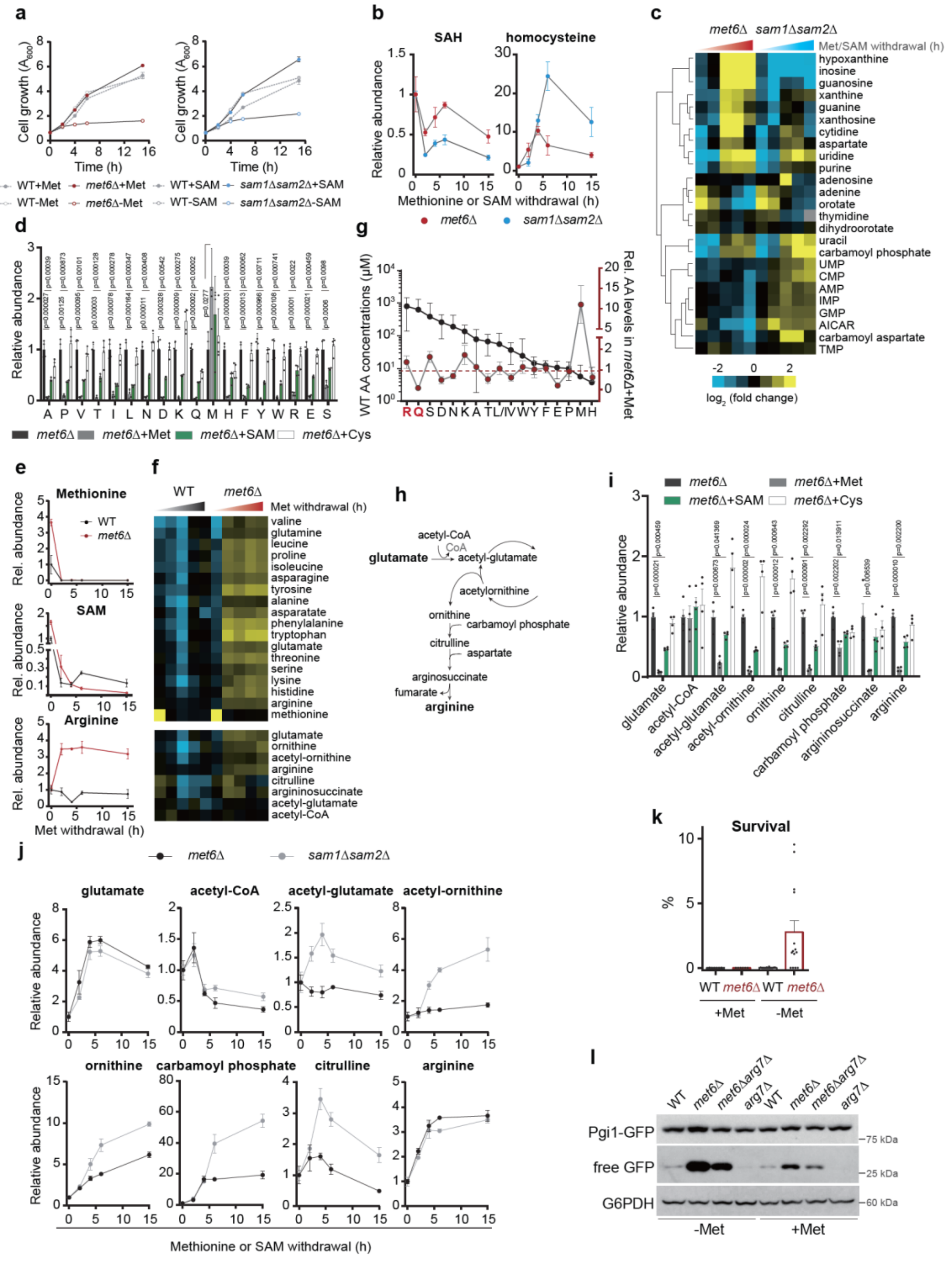

Supplementary Fig. 1: Cellular SAM depletion activates nitrogen anabolism.
(**a**) Growth curves of WT, *met6Δ*, and *sam1Δsam2Δ* cells. Data are represented as mean ± SD (n = 3, n=biologically independent samples). (**b**) Relative abundances of SAH and homocysteine in *met6Δ* and *sam1Δsam2Δ* cells under respective methionine or SAM starvation. Data are represented as mean ± SD (n = 3, n=biologically independent samples). (**c**) Heatmap depicting abundances of metabolites in nucleotide metabolism in *met6Δ* and *sam1Δsam2Δ* cells under respective methionine or SAM starvation. (**d**) Relative abundances of amino acids in *met6Δ* cells 4 h after the switch

to minimal medium with or without 1 mM of methionine, SAM, or cysteine. Data are represented as mean ± SD (n = 4, n=biologically independent samples). (**e**) Relative abundances of methionine, SAM, and arginine in WT and *met6Δ* cells. Data are represented as mean ± SD (n=3, n=biologically independent samples). (**f**) Heatmap depicting abundances of amino acids and arginine pathway metabolites in WT and *met6Δ* cells under methionine starvation. (**g**) Absolute concentrations of amino acids in WT cells logarithmically growing in SD medium (black) and their relative abundances in *met6Δ* cells grown with 1 mM methionine (red). Data are represented as mean ± SD (n=10, n=biologically independent experiments). (**h**) The arginine biosynthesis pathway. (**i**) Relative abundances of amino acids in *met6Δ* cells 4 h after the switch to minimal medium with or without 1 mM of methionine, SAM, or cysteine. Data are represented as mean ± SD (n = 4, n=biologically independent samples). (**j**) Relative abundances of arginine pathway metabolites in *met6Δ* and *sam1Δsam2Δ* cells over the periods of their respective starvation. Data are represented as mean ± SD (n=3, n=biologically independent samples). (**k**) The survival of WT and *met6Δ* under MR. Data are represented as mean ± SD (n = 6, n=biologically independent samples). (**l**) Autophagy was assayed by the GFP cleavage of Pgi1-GFP in WT, *met6Δ*, *arg7Δ*, and *met6Δarg7Δ* under MR. Data are representative of two independent experiments. Statistical analysis in this figure was performed using two-tailed Student's t test. Source data are provided as a Source Data file.

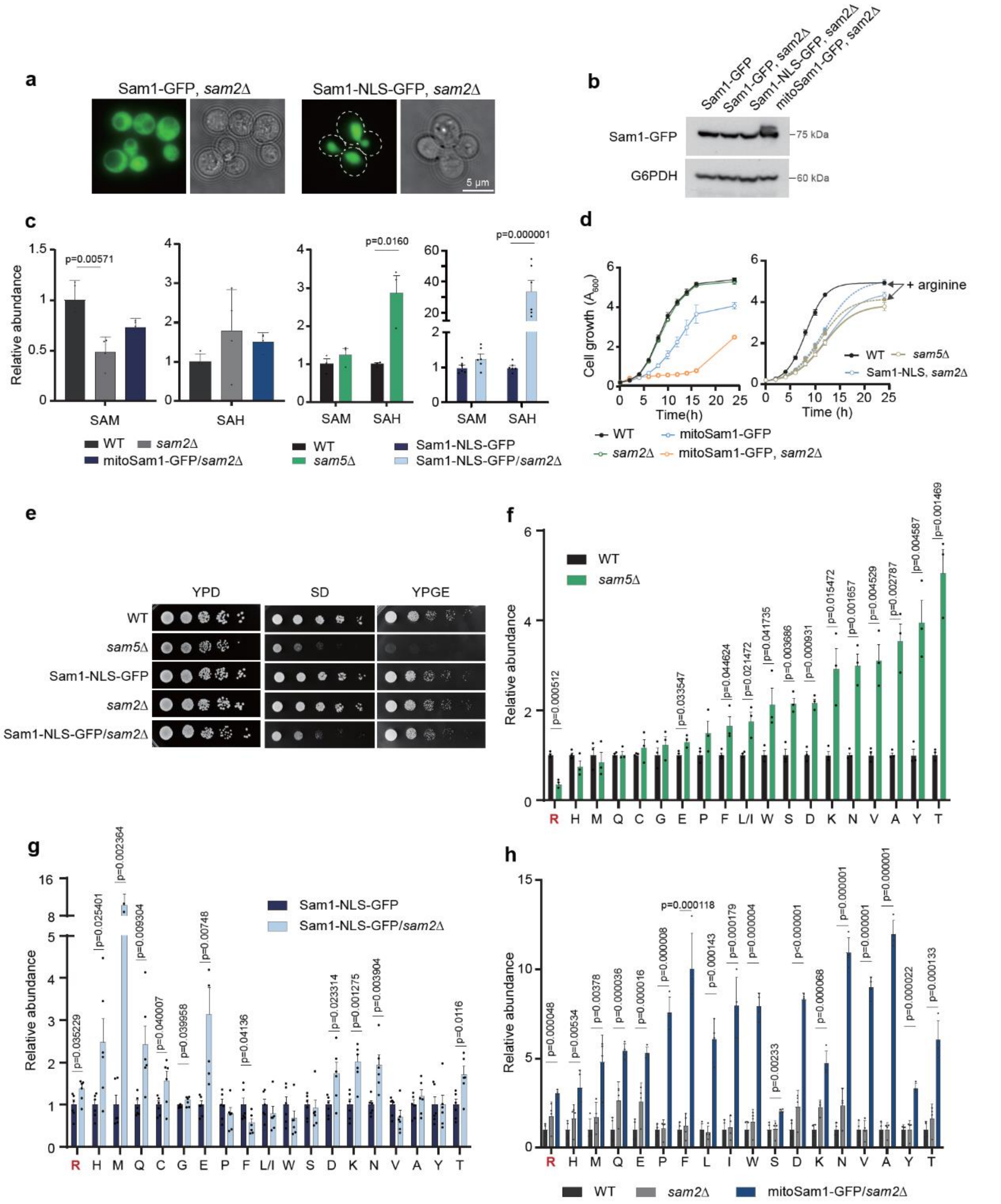

Supplementary Fig. 2: Imbalanced cellular amino acid pools and growth defects in the mutants manipulating the source of mitochondrial SAM.

(**a**) Localization of Sam1-GFP and Sam1-NLS-GFP in the *sam2Δ* mutant. (**b**) Protein levels of Sam1-GFP in indicated strains. Data are representative of two independent experiments. (**c**) Relative abundances of SAM and SAH in WT and indicated mutants. Data are represented as mean ± SD (n=4, n=biologically independent samples). (**d**) Growth curves of WT and the indicated mutants in SD medium. (**e**) Growth of WT and the indicated mutants on YPD, SD, and YPGE (glycerol and ethanol) plates. Data are represented as mean ± SD (n=4, n= biologically independent samples). (**f-h**) Relative abundances of amino acids in (**f**) WT and *sam5Δ* cells, (**g**) WT and *sam2Δ* strains where Sam1 is directed into the nucleus (Sam1-NLS-GFP), and (**h**) WT, *sam2Δ,* and

mitoSam1-GFP/*sam2Δ* strains. Cells were harvested in the log phase. Data are represented as mean ± SD (**f**, n = 3; **g**, n=6; **h**, n=4; n=biologically independent samples). Statistical analysis in this figure was performed using two-tailed Student's t test. Source data are provided as a Source Data file.

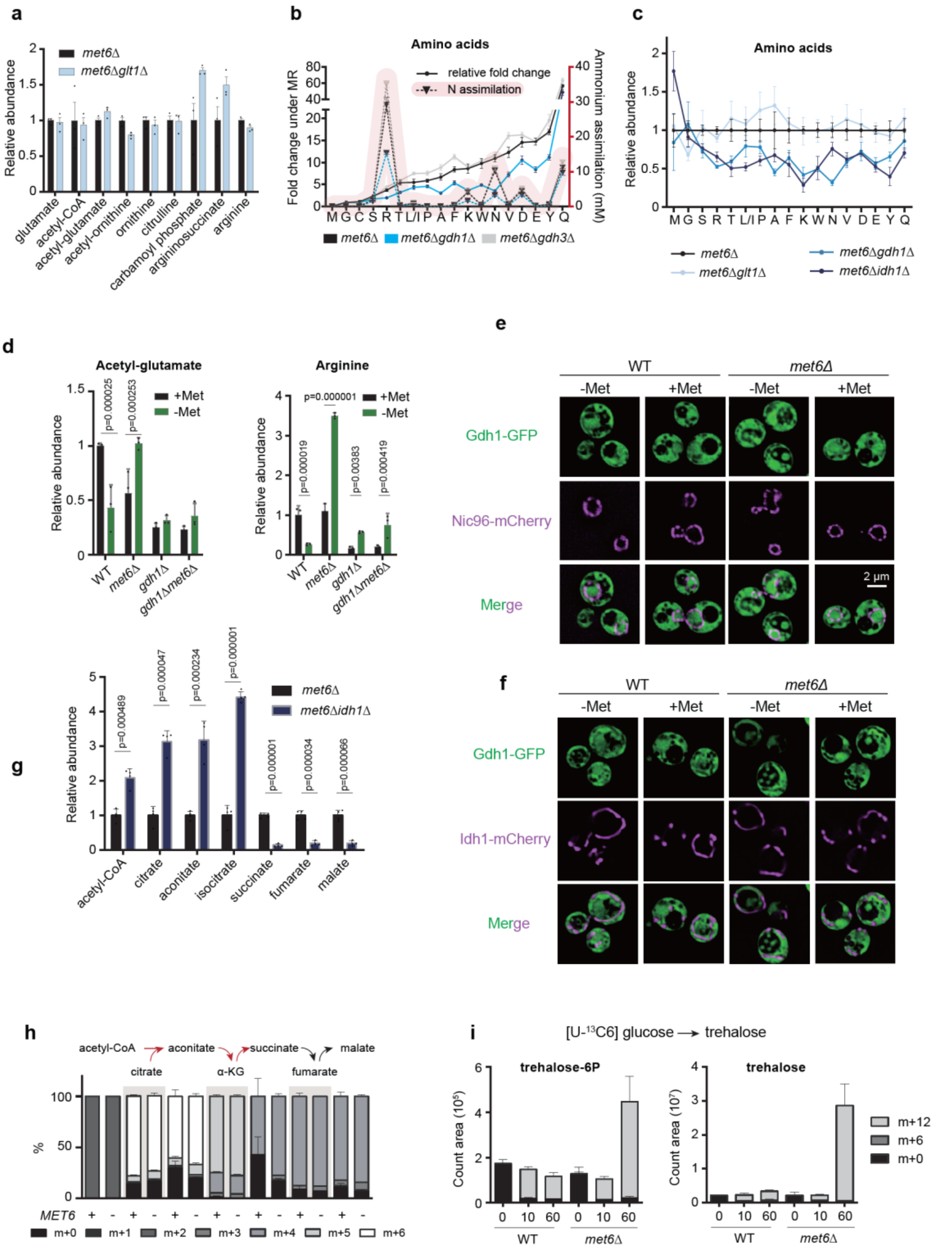

Supplementary Fig.3: Glucose-derived acetyl-CoA and αKG exit the TCA cycle to support arginine synthesis upon MR.

(**a**) Relative abundances of metabolites in the arginine biosynthesis pathway in the indicated mutants after 4 h MR. Data are represented as mean ± SD (n = 3, n=biologically independent samples). (**b**) Relative abundances of amino acids and absolute amounts of ammonia absorbed by each amino acid after 4 h MR. Data are represented as mean ± SD (n = 3, n=biologically independent samples). (**c**) Relative

abundances of amino acids in indicated cells after 4 h MR. Data are represented as mean ± SD (*met6Δ*, n=7; *met6Δglt1Δ*, n=3; *met6Δgdh1Δ*, n=3; *met6Δidh1Δ*, n=4; n=biologically independent samples). (**d**) Relative abundances of acetyl-glutamate and arginine in WT, *met6Δ*, *gdh1Δ*, and *gdh1Δmet6Δ* cells in SD medium with 1mM methionine and 4 h after MR. Data are represented as mean ± SD (n = 3, n=biologically independent samples). Statistical analysis was performed using two-tailed Student's t test. (**e-f**) Localization of Gdh1-GFP in WT and *met6Δ* cells with or without MR. The nuclear pore complex protein Nic96 and the mitochondrial matrix protein Idh1 were mCherry-tagged. Data are representative of at least two independent experiments. (**g**) Relative abundances of TCA cycle metabolites after 4 h MR. Data are represented as mean ± SD (n = 4, n=biologically independent samples). Statistical analysis was performed using two-tailed Student's t test. (**h**) The percent abundance of ^{13}C-labeled TCA metabolites in WT and *met6Δ* cells after 60 min MR. Data are represented as mean ± SD (n = 4, n=biologically independent samples). (**i**) Spectra intensities of ^{13}C-labeled and unlabeled trehalose-6-phosphate and trehalose in WT and *met6Δ* cells challenged by MR in [U-$^{13}C6$] glucose tracing media. Data are represented as mean ± SD (n = 4, n=biologically independent samples). Source data are provided as a Source Data file.

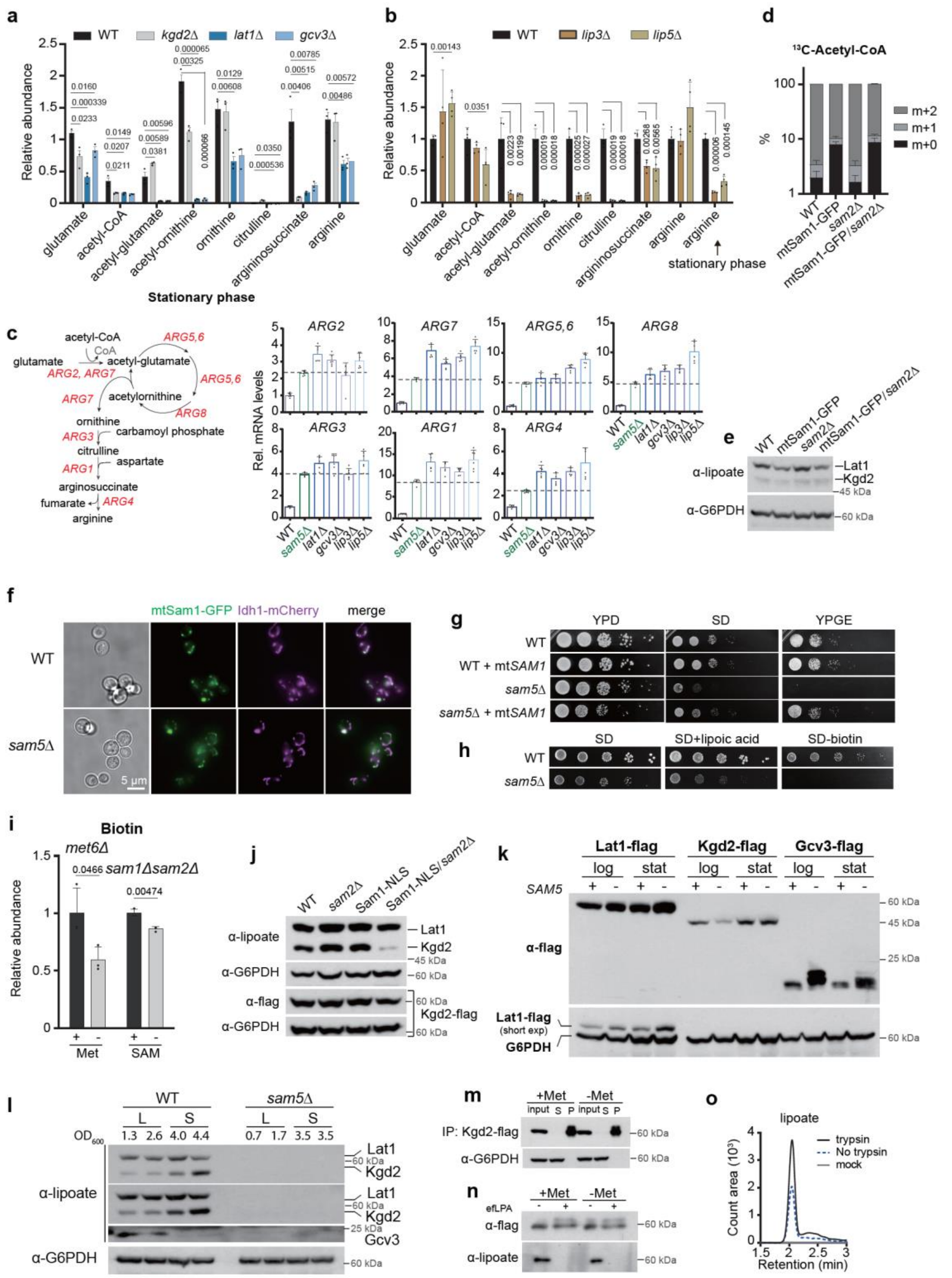

Supplementary Fig. 4: Lipoylation is a sentinel modification responding to cellular SAM decrease to regulate arginine synthesis.

(**a-b**) Relative abundances of arginine metabolites in indicated cells. Data are represented as mean ± SD (4a, n = 3, n=biologically independent samples; 4b, n = 4, n=biologically independent samples). (**c**) Left: Arginine biosynthesis pathway; Right: mRNA levels of arginine biosynthetic genes in WT and indicated mutants in the logarithmic phase. Data are represented as mean ± SD (n = 6, n=biologically

independent samples). (**d**) The percent abundance of ^{13}C-labeled acetyl-CoA in WT and indicated mutant cells after 10 min of ^{13}C tracing. Data are represented as mean ± SD (n = 4, n=biologically independent samples). (**e**) Western blots assaying protein lipoylation of Lat1 and Kgd2 in WT and the mutant cells in the logarithmic phase. Data are representative of two independent experiments. (**f**) Fluorescence imaging of mtSam1-GFP localization in indicated cells. Mitochondria were visualized by Idh1-mCherry. Data are representative of two independent experiments. (**g-h**) Growth of indicated strains on indicated growth plates. (**i**) Biotin levels in *met6Δ* and *sam1Δsam2Δ* cells 2 h after cellular SAM deprivation. Data are represented as mean ± SD (n = 3, n=biologically independent samples). (**j**) Western blots assaying protein lipoylation and Kgd2-flag levels in cells in the stationary phase. Data are representative of at least three independent experiments. Note that Kgd2 was not flag-tagged in the strains used for assaying lipoylation. (**k**) Western blots assaying Lat1-flag, Kgd2-flag, and Gcv3-flag protein abundances. Data are representative of at least three independent experiments. (**l**) Western blots assaying protein lipoylation in WT and *sam5Δ* cells in different phases. L: log phase; S: stationary phase. (**m**) Immunoprecipitation (IP) of Kgd2-flag. S: supernatant; P: pellet. Data are representative of two independent experiments. (**n**) Lipoamidase treatment of IPed Kgd2. Data are representative of two independent experiments. (**o**) Trypsin treatment before Lipoamidase treatment increased the release of lipoic acid. Data are representative of at least three independent experiments. Statistical analysis in this figure was performed using two-tailed Student's t test. Source data are provided as a Source Data file.

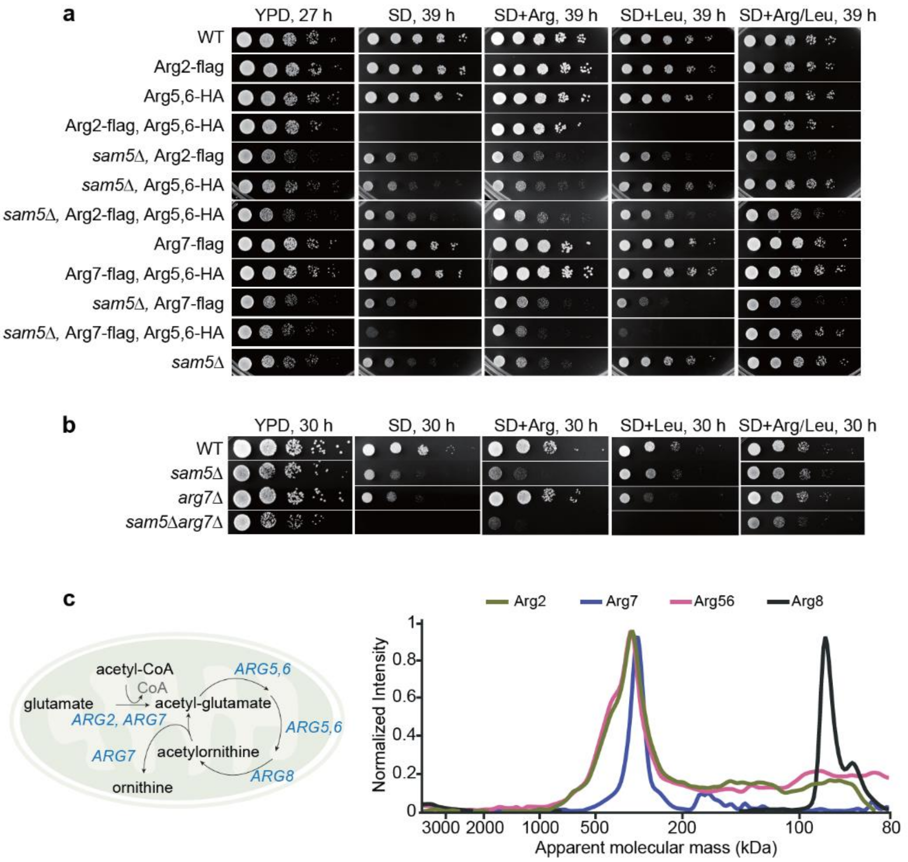

Supplementary Fig. 5: *SAM5* deletion alters arginine metabolon that averts leucine biosynthesis.

(**a-b**) Growth of the indicated strains on YPD, SD, and SD containing either 1 mM arginine, 1 mM leucine, or 1 mM of both. Note that Supplementary Fig. 5a was the same spotting experiment shown in Fig. 5d, with the controls indicating that the single gene with C-terminal tagging did not affect cell growth. Data are representative of at least three independent experiments. (**c**) Left: The acetyl cycle with genes responsible for each step; Right: The evidence supports the metabolon assembly of Arg2, Arg7, and Arg5,6 from the mitochondrial complexome study. Source data are provided as a Source Data file.

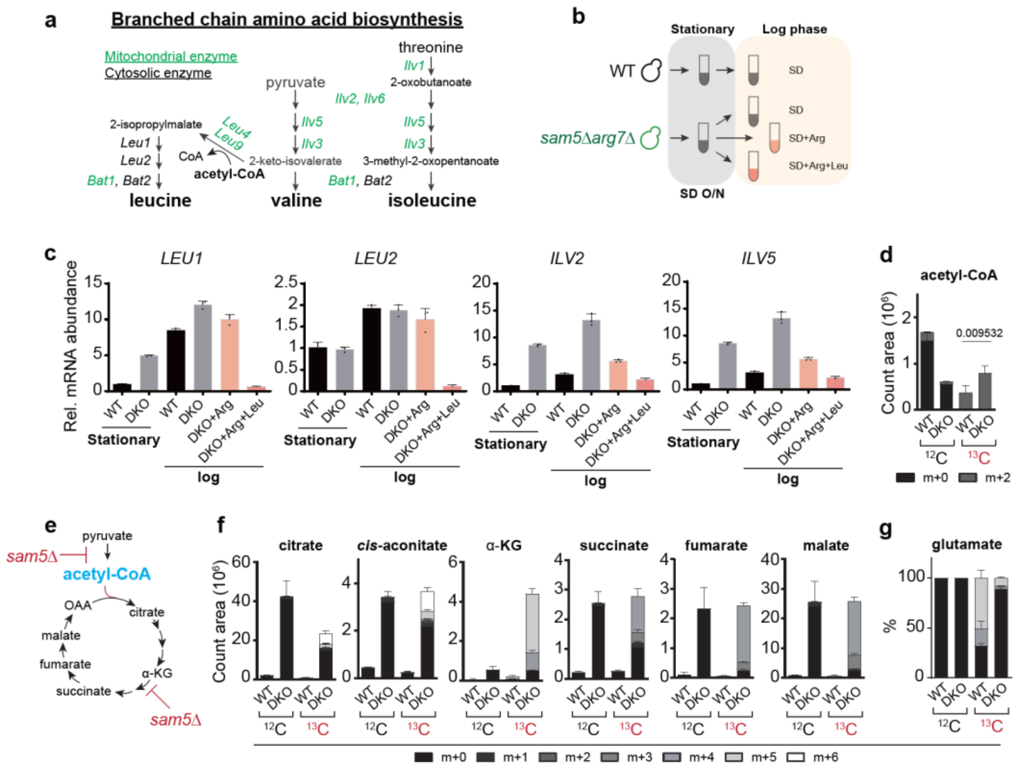

Supplementary Fig. 6: The hierarchy of acetyl-CoA allocation in mitochondrial metabolism.

(**a**) Schematic of BCAA biosynthesis. The mitochondrial enzymes are highlighted in green. (**b**) Schematic of the experiment design for measuring mRNA transcript levels of leucine genes in WT and *sam5Δarg7Δ* cells. O/N, overnight culture. (**c**) Relative mRNA transcript levels of *LEU1*, *LEU2*, *ILV2*, and *ILV5* in WT and *sam5Δarg7Δ* (DKO) cells. Data are represented as mean ± SD (n=3, n=biologically independent samples). (**d**) Spectra intensities of labeled and unlabeled acetyl-CoA in WT and *sam5Δarg7Δ* (DKO) cells. Data are mean ± SD (n = 4, n=biologically independent samples). Statistical analysis was performed using two-tailed Student's t test. (**e**) Schematic of the TCA cycle. The acetyl-CoA step and the steps inhibited by *sam5Δ* are highlighted. (**f**) Spectra intensities of isotopic metabolites in the TCA cycle. ^{12}C: before ^{13}C tracing; ^{13}C: 1 h after ^{13}C tracing. Data are mean ± SD (n = 4, n=biologically independent samples). (**g**) The percent abundance of ^{13}C-labeled glutamate. Data are represented as mean ± SD (n = 4, n=biologically independent samples). Source data are provided as a Source Data file.

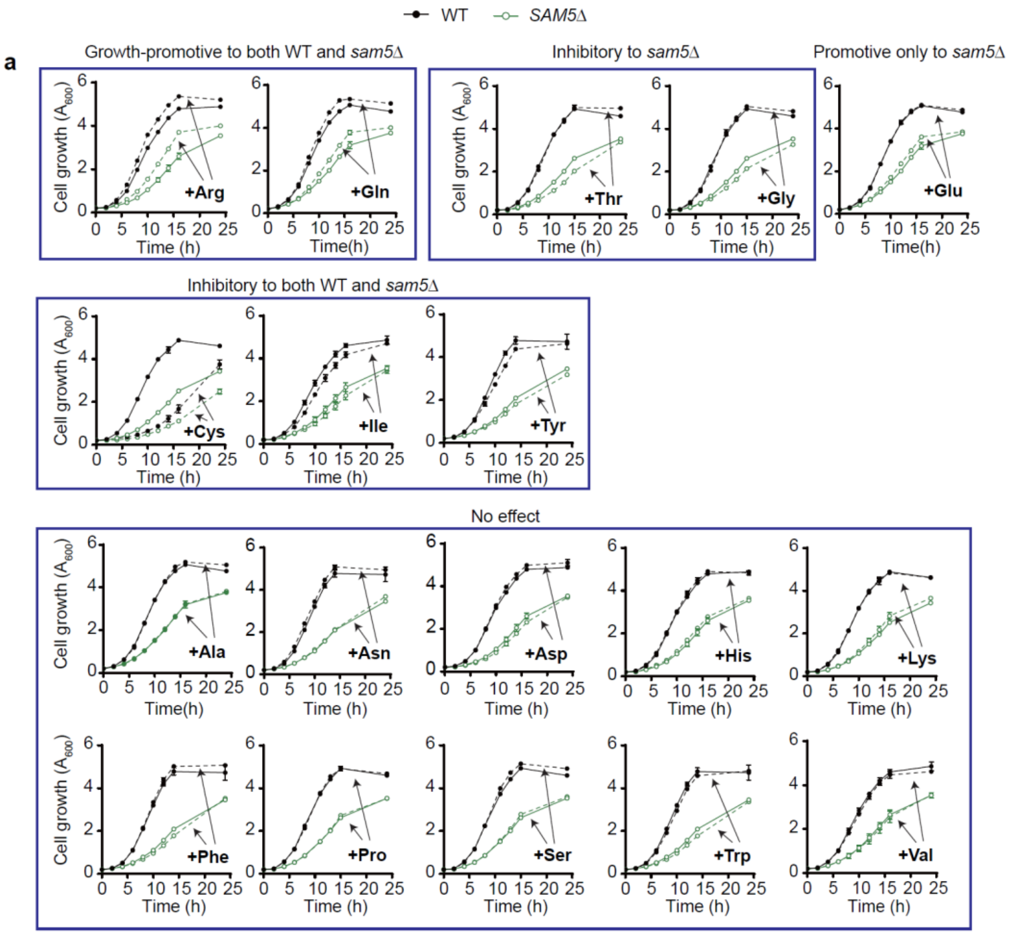

Supplementary Fig.7: Methionine and leucine impose Sam5-dependent growth control.

(**a**) Growth curves of WT and *sam5Δ* cells with or without 1 mM supplementation of an indicated amino acid. Note that growth curves with methionine and leucine are shown in Fig. 7a. Data are represented as mean ± SD (n=4, n=biologically independent samples).

Supplementary Table 1: KEGG pathway analysis of *sam1Δsam2Δ* and *met6Δ* strains.

	sam1Δsam2Δ				*met6Δ*			
KEGG pathway	2 h	4 h	6 h	15 h	2 h	4 h	6 h	15 h
**Alanine, aspartate and glutamate metabolism**	0.0003	0.0000	0.0010	0.0036	0.0189	0.0008	0.0003	0.0002
**Arginine biosynthesis**	0.0010	0.00000036	0.000024	0.0054	0.0091	0.0011	0.0004	0.0002
**Arginine and proline metabolism**	0.7098	0.0590	0.0395	0.0907	0.3470	0.0095	0.0561	0.0127
**beta-Alanine metabolism**	0.0127	0.0633	0.1003	0.6831	0.0136	0.0927	0.3646	0.3357
**Cysteine and methionine metabolism**	0.0402	0.0064	0.0073	0.0406	0.0026	0.1142	0.1787	0.0636
**Glycine, serine and threonine metabolism**	0.4574	0.1388	0.1107	0.6281	0.2011	0.0978	0.1491	0.4587
**Lysine biosynthesis**	0.1744	0.0090	0.0199	0.2037	0.0388	0.0709	0.0999	0.0834
**Nitrogen metabolism**	0.2169	0.0633	0.0890	0.0790	0.0211	0.0840	0.1031	0.0925
**Phenylalanine, tyrosine and tryptophan biosynthesis**	0.6454	0.0298	0.1722	0.3433	0.0778	0.1579	0.0251	0.3982
**Purine metabolism**	0.0004	0.6471	0.1057	0.0050	0.3576	0.0417	0.0849	0.0107
**Pyrimidine metabolism**	0.0191	0.0229	0.0061	0.0138	0.0045	0.0492	0.0830	0.0229
**Valine, leucine and isoleucine biosynthesis**	0.2458	0.0243	0.0504	0.0392	0.2544	0.0445	0.0197	0.0545

Note: p value indicates the significance of the pathway under conditions where the indicated strains were subject to methionine or S-adenosylmethionine starvation. Statistical analysis was performed using one-tailed hypergeometric test.
