# Supplementary material for: Methionine restriction constrains lipoylation and activates mitochondria for nitrogenic synthesis of amino acids
Source: Nat Commun. 2023 May 2;14:2504. doi: 10.1038/s41467-023-38289-9 (PMC10154411; doi:10.1038/s41467-023-38289-9)
Supplement: Supplementary file 3 — Description of Additional Supplementary Files [file 41467_2023_38289_MOESM3_ESM.pdf]

Description of Additional Supplementary Files:

Supplementary Data 1: A list of yeast strains used in this study.

Supplementary Data 2: A list of primers used in this study.
