# Supplementary material for: Methionine restriction constrains lipoylation and activates mitochondria for nitrogenic synthesis of amino acids
Source: Nat Commun. 2023 May 2;14:2504. doi: 10.1038/s41467-023-38289-9 (PMC10154411; doi:10.1038/s41467-023-38289-9)
Supplement: Supplementary file 6 — Reporting Summary [file 41467_2023_38289_MOESM6_ESM.pdf]

nature portfolio

Corresponding author(s): Cunqi Ye

Last updated by author(s): Apr 10, 2023

Reporting Summary

Nature Portfolio wishes to improve the reproducibility of the work that we publish. This form provides structure for consistency and transparency in reporting. For further information on Nature Portfolio policies, see our Editorial Policies and the Editorial Policy Checklist.

Statistics

For all statistical analyses, confirm that the following items are present in the figure legend, table legend, main text, or Methods section.

n/a	Confirmed	
☐	☒	The exact sample size (*n*) for each experimental group/condition, given as a discrete number and unit of measurement
☐	☒	A statement on whether measurements were taken from distinct samples or whether the same sample was measured repeatedly
☐	☒	The statistical test(s) used AND whether they are one- or two-sided<br>*Only common tests should be described solely by name; describe more complex techniques in the Methods section.*
☒	☐	A description of all covariates tested
☒	☐	A description of any assumptions or corrections, such as tests of normality and adjustment for multiple comparisons
☐	☒	A full description of the statistical parameters including central tendency (e.g. means) or other basic estimates (e.g. regression coefficient) AND variation (e.g. standard deviation) or associated estimates of uncertainty (e.g. confidence intervals)
☐	☒	For null hypothesis testing, the test statistic (e.g. *F*, *t*, *r*) with confidence intervals, effect sizes, degrees of freedom and *P* value noted<br>*Give P values as exact values whenever suitable.*
☒	☐	For Bayesian analysis, information on the choice of priors and Markov chain Monte Carlo settings
☒	☐	For hierarchical and complex designs, identification of the appropriate level for tests and full reporting of outcomes
☒	☐	Estimates of effect sizes (e.g. Cohen's *d*, Pearson's *r*), indicating how they were calculated

Our web collection on statistics for biologists contains articles on many of the points above.

Software and code

Policy information about availability of computer code

Data collection	Intracellular metabolites were measured using the QTRAP 6500+ System, ABSCIEX. The raw data were extracted with the software Analyst v1.7.2 and OS v1.7 from SCIEX;<br>Real-time PCR was performed in a Bio-Rad CFX96 Connect device;<br>All microscopy images were taken under a 100X, 1.4 NA oil-immersion objective lens with a Deltavision Elite microscope;<br>The bioreactor used in this study to perform YMC experiments is from INFORS (Model minifors 2, 3 L).
Data analysis	The normalized abundances of metabolites were log-transformed, centered about the median, and clustered by Spearman rank correlation algorithm using Cluster 3, and heat maps were obtained by the software Treeview 1.2.0. MetaboAnalyst 5.0, a web-based analysis platform68 for KEGG pathway analysis;<br>ImageJ v1.8.0 (National Institutes of Health) was used for Western blot densitometry analysis.

For manuscripts utilizing custom algorithms or software that are central to the research but not yet described in published literature, software must be made available to editors and reviewers. We strongly encourage code deposition in a community repository (e.g. GitHub). See the Nature Portfolio guidelines for submitting code & software for further information.

Data

Policy information about availability of data

All manuscripts must include a data availability statement. This statement should provide the following information, where applicable:

- Accession codes, unique identifiers, or web links for publicly available datasets
- A description of any restrictions on data availability
- For clinical datasets or third party data, please ensure that the statement adheres to our policy

All data supporting the findings of this work are available within the article, supplementary information, and source data. Targeted metabolic profiling raw data generated in this study have been deposited in Metabolomics Workbench under Project ID: PR001637 (Project DOI: doi: 10.21228/M83M7P). Source Data are provided with this paper.

Human research participants

Policy information about studies involving human research participants and Sex and Gender in Research.

Reporting on sex and gender	N/A
Population characteristics	N/A
Recruitment	N/A
Ethics oversight	N/A

Note that full information on the approval of the study protocol must also be provided in the manuscript.

Field-specific reporting

Please select the one below that is the best fit for your research. If you are not sure, read the appropriate sections before making your selection.

- [x] Life sciences
- [ ] Behavioural & social sciences
- [ ] Ecological, evolutionary & environmental sciences

For a reference copy of the document with all sections, see nature.com/documents/nr-reporting-summary-flat.pdf

Life sciences study design

All studies must disclose on these points even when the disclosure is negative.

Sample size	Sample size was chosen based on experimental variability and the requirement of experimental samples for statistical analysis. At least three biological replicates per group were collected to perform statistical testing, which is sufficient for a minimal data set underlying the figures provided in the paper in order to support and interpret our central findings. Detailed n is indicated in the figure legends. No statistical method was used to determine the sample size.
Data exclusions	No data or samples were excluded from the analysis.
Replication	Experiments were performed at least twice to make sure that the results are reproducible, and representative data are shown. All attempts at replication were successful. Details are shown in Method.
Randomization	All samples were randomly allocated to experimental groups and processed.
Blinding	For the analyses of the qPCR data and cellular metabolites, blinding was not necessary since these analyses were observer independent. Blinding was performed during the acqusition of the metabolic raw data. The researcher who inspected the spectra intensities were blinded to the sample information. Western blotting was not performed blind to load samples by order. Fluorescence microscopy was not performed blind as for a validation of protein localization with the known organelle marker protein.

Reporting for specific materials, systems and methods

We require information from authors about some types of materials, experimental systems and methods used in many studies. Here, indicate whether each material, system or method listed is relevant to your study. If you are not sure if a list item applies to your research, read the appropriate section before selecting a response.

Materials & experimental systems

n/a	Involved in the study	
☐	☒	Antibodies
☐	☒	Eukaryotic cell lines
☒	☐	Palaeontology and archaeology
☒	☐	Animals and other organisms
☒	☐	Clinical data
☒	☐	Dual use research of concern

Methods

n/a	Involved in the study	
☒	☐	ChIP-seq
☒	☐	Flow cytometry
☒	☐	MRI-based neuroimaging

Antibodies

Antibodies used: mouse anti-FLAG M2 antibody (Sigma, Cat# F3165, 1:5000), rabbit anti-G6PDH (Sigma, Cat#A9521, 1:5000), rabbit anti-lipoic aicd (Milipore, Cat#437695, 1:3000)

Validation: All antibodies are commercially available and were validated as follows:
https://www.sigmaaldrich.cn/CN/zh/product/sigma/f3165
https://www.sigmaaldrich.cn/CN/zh/product/sigma/a9521
https://www.merckmillipore.com/CN/zh/product/Anti-Lipoic-Acid-Rabbit-pAb,EMD_BIO-437695

Eukaryotic cell lines

Policy information about cell lines and Sex and Gender in Research

Cell line source(s): The prototrophic CEN.PK strain of the budding yeast S. cerevisiae background was used in all experiments.

Authentication: None of the cell lines used were authenticated.

Mycoplasma contamination: The yeast cells were not tested for mycoplasma.

Commonly misidentified lines (See ICLAC register): No commonly misidentified cell lines were involved in this study.
